# Heparin inhibits intracellular *Mycobacterium tuberculosis* bacterial replication by reducing iron levels in human macrophages

**DOI:** 10.1038/s41598-018-25480-y

**Published:** 2018-05-08

**Authors:** Rodrigo Abreu, Lauren Essler, Allyson Loy, Frederick Quinn, Pramod Giri

**Affiliations:** 1 Department of Infectious Diseases, College of Veterinary Medicine, University of Georgia, Athens, GA 30602 USA; 2 Department of Microbiology, University of Georgia, Athens, GA 30602 USA

## Abstract

Iron is a crucial micronutrient for both mammals and their associated pathogens, and extensive literature has shown that *Mycobacterium tuberculosis* (Mtb) bacilli inhibited from acquiring iron from the host are severely attenuated. In contrast, increased dietary iron concentrations or patients with hemochromatosis have long been associated with a more severe tuberculosis (TB) disease outcome. We have observed that upon macrophage infection, Mtb bacilli strongly promote intracellular iron sequestration, both through increased expression of hepcidin, a key mammalian iron regulatory protein, and downregulation of the iron exporter protein, ferroportin. Heparin is a highly sulfated glycosaminoglycan released by mast cells and basophils at sites of tissue injury. During Mtb infection, heparin alters intracellular trafficking in alveolar epithelial cells and decreases extrapulmonary dissemination but recently, heparin also has been reported to inhibit hepcidin expression in hepatocytes, decreasing intracellular iron availability. In this report, we demonstrate that heparin significantly reduces hepcidin expression in macrophages infected with Mtb bacilli. Heparin-treated macrophages have higher ferroportin expression compared to untreated macrophages, promoting iron export and decreasing iron availability to intracellular bacilli. Thus, here we describe a novel immunomodulatory effect and potential therapeutic role for heparin against mycobacterial infection in human macrophages.

## Introduction

*Mycobacterium tuberculosis*, the causative agent of tuberculosis (TB), infects nearly 10 million people annually and causes approximately 1.5 million fatalities globally. Despite extensive efforts to control and eradicate TB, we are still failing to meet the milestones of the WHO *End TB strategy*. One-third of the world population is estimated to be latently infected with Mtb with a 10% lifetime risk of reactivation. However, for immunocompromised patients the risk increases to a 10% chance of disease progression every year^1^.

*Mycobacterium tuberculosis* is one of the most prevalent human pathogens that has evolved to persist in alveolar macrophages ultimately causing extensive lung inflammation and pathology^2,3^. Macrophages serve as the major intracellular niche for Mtb. Upon successful infection, Mtb bacilli evade the macrophage innate antimicrobial functions, inhibit the phagolysosome fusion process and gain access to crucial intracellular nutrients^4^. Inhibition of the inflammasome and impaired IL-1β secretion is associated with increased intracellular bacterial proliferation^5^. Alternatively, chelation of intracellular nutrients such as iron strongly inhibits Mtb replication in macrophages^6,7^. Iron dysregulation has been strongly associated with worsened disease outcomes in Mtb infected patients^8^, while effective iron export in macrophages decreased intracellular mycobacterial replication^9^.

Heparin is a highly sulfated glycosaminoglycan released by mast cells and basophils at sites of tissue injury. Despite its well-described anticoagulant activity, heparin’s physiological role in innate immunity during infection is not fully understood^10^. The mycobacterial adhesin heparin-binding hemagglutinin (HBHA) is an important virulence factor for adhesion, internalization and dissemination from the lung during Mtb infection^11,12^. Heparin and other glycosaminoglycans can decrease the *Mtb* bacterial burden in epithelial cells, but its impact in intracellular replication in macrophages has not yet been investigated. Heparin has multiple modulatory effects on the host cells^13^. For example, heparin has been implicated in multiple biological processes and is capable of interacting with hundreds of human proteins^14,15^. As an immunomodulatory molecule, heparin has been shown to inhibit complement activation, modify cytokine secretion in human mononuclear cells and inhibit leukocyte recruitment^16–19^. Heparin also has been reported to have some antiviral activity through direct interaction with viral proteins^20,21^.

Most studies with heparin have been performed on hepatocytes, where the glycosaminoglycan has been shown to inhibit hepcidin expression, thereby decreasing intracellular iron levels in this iron regulatory cell type^22–25^. Interestingly, we have now observed that upon macrophage infection, Mtb bacilli strongly promote intracellular iron sequestration both through induction of hepcidin and direct down-regulation of the iron exporter ferroportin (unpublished data).

In this study, we report that heparin significantly inhibits hepcidin expression in human macrophages after mycobacterial infection. Heparin-treated macrophages express higher ferroportin surface levels compared to untreated controls, promoting iron export and decreasing iron availability to intracellular bacilli. Similar to iron-chelation treatment, heparin significantly reduces Mtb intracellular replication in macrophages. Bacterial internalization and intracellular viability rates were similar between the heparin-treated and control infections, thus the observed lower replication rate is likely the result of the inability of the intracellular bacilli to sequester iron from their niche.

This study suggests a new immunomodulatory function of heparin in macrophages, and a possible protective mechanism for sulfated glycosaminoglycan during Mtb infection. The outcome of this study also provides impetus for screening and assess of modified heparins as novel immunomodulatory anti-mycobacterial therapeutic molecules.

## Results

### Heparin decreases mycobacterial intracellular replication in human macrophages

**Figure 1:**
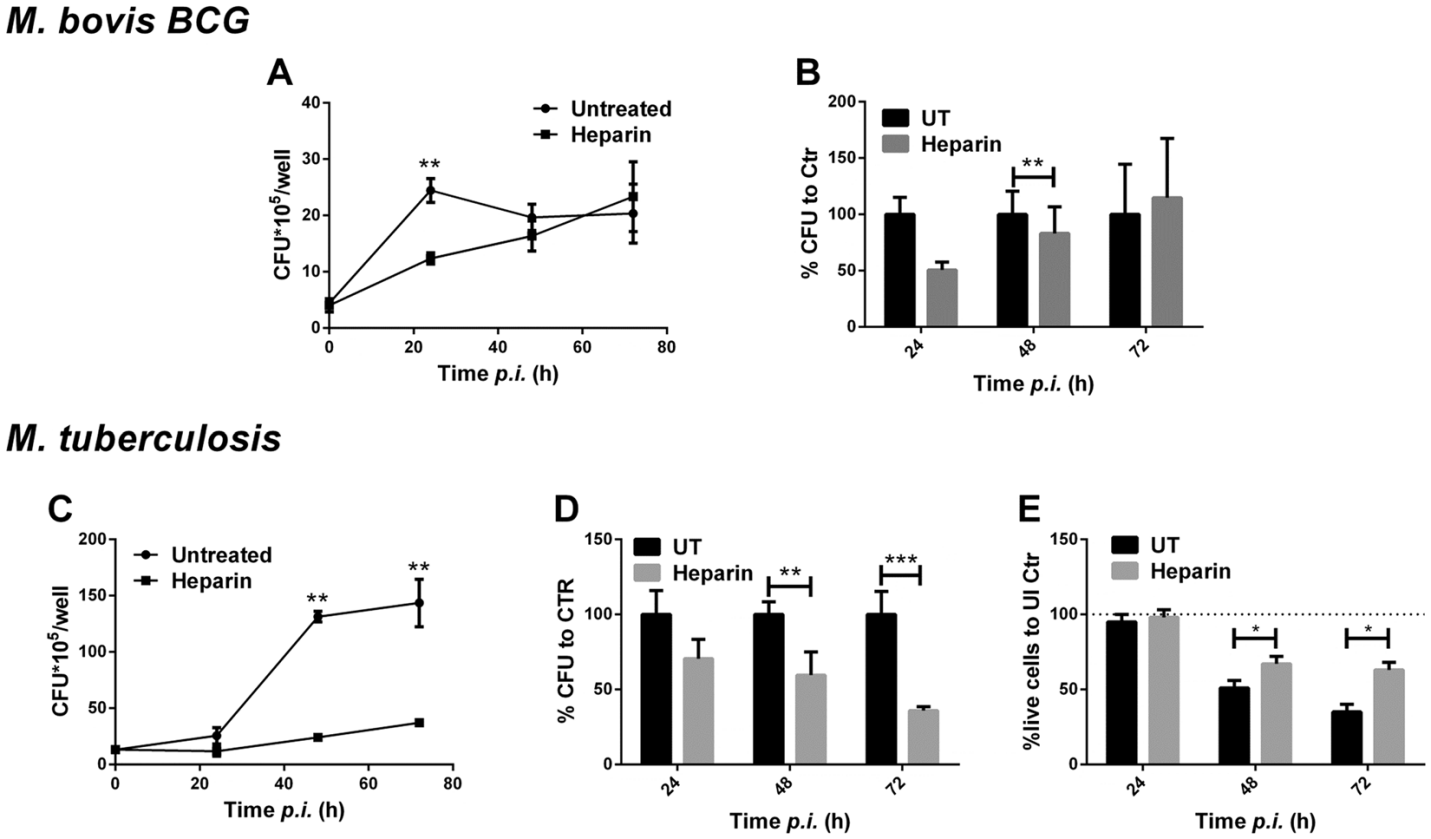
Heparin inhibits Mtb and BCG replication. (**A**,**C**) Intracellular CFU in THP-1 macrophages infected with BCG (**A**) and Mtb (**C**) at an MOI of 10 after 16 hours of treatment with 50 µg heparin. (**B**,**D**) Percentage of intracellular bacilli in heparin treated macrophages at 24, 48 and 72 hours post infection with BCG (**B**) and Mtb (**D**). (**E**) Trypan blue exclusion cell viability in Mtb infected-THP-1 macrophages at 24, 48 and 72 hours post-infection. For a and c macrophages were seeded in 12 well plates Data from three independent experiments. * *p* < *0*.05, **p < 0.01, ***p < 0.001.

Heparin-binding hemaglutinin protein (HBHA) may be an important adhesin for effective attachment of Mtb bacilli to alveolar epithelial cells^12^. However, other than binding to HBHA and interfering with attachment to these epithelial cells, the roles for heparin and other sulfated glycosaminoglycans in *Mtb* intracellular replication and survival have not been tested. *Mycobacterium bovis* BCG is an avirulent vaccine strain frequently used as a BSL2 model to study Mtb replication in macrophages. To assess the impact of heparin on BCG internalization and intracellular replication, THP-1 macrophages were treated overnight with 50 µg/ml (≈10U/ml) heparin and infected with opsonized BCG at a MOI of 10. After two hours, bacterial uptake was similar between the heparin-treated and untreated macrophages (p = 0.792); however, by 24 hours post infection, heparin-treated macrophages showed a significant 50.6% (±6.97) reduction in intracellular bacterial numbers when compared to untreated controls (p = 0.006, Fig. 1A and B). Because BCG is an avirulent strain of *M*. *bovis*, intracellular replication is limited compared to fully virulent strains in human macrophages. Thus, after 48 hours, intracellular replication stops for control BCG infections, unrelated to the heparin treatment (Fig. 1B).

Considering the reduction in early intracellular BCG replication, we assessed and compared the impact of heparin treatment on Mtb-infected macrophages. As observed with BCG, heparin-treated and control macrophages showed no differences in Mtb uptake two hours after internalization (*p* = 0.556, Fig. 1C). However, by 48 hours post infection, heparin-treated macrophages showed significantly decreased intracellular bacterial burdens compared to untreated controls (*p* = 0.045, Fig. 1C and D). By 72 hours post infection, heparin treatment continued to decrease the intracellular bacterial burden compared with untreated controls, reaching a 64% (±5.807) decrease (Fig. 1D). Furthermore, heparin treatment significantly improved macrophage cell viability by 72 hours after Mtb infection (mean difference was 28 ± 4.082%, Fig. 1E). Altogether, these data point to a host-protective role of heparin during Mtb infection by limiting intracellular bacterial burden in macrophages.

### Heparin treatment does not affect bacterial viability *in vitro*

**Figure 2:**
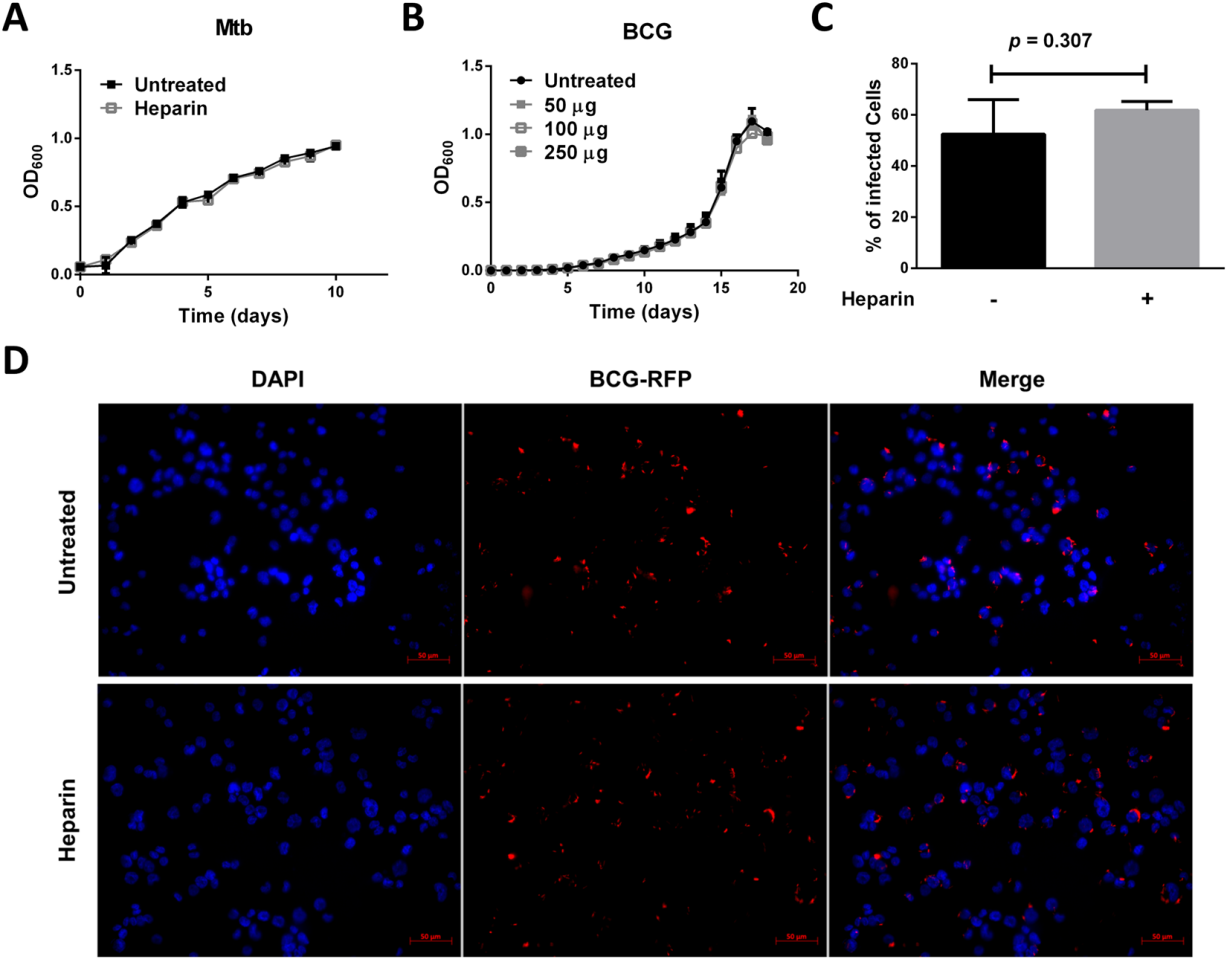
Heparin has no direct impact in bacterial growth or macrophage internalization. (**A**,**B**) BCG (**A**) and Mtb (**B**) growth in heparin-supplemented 7H9 medium. (**C**) Percentage of infected cells in 9 random fields (10x) from 3 independent experiments as represented in (**B**). (**D**) BCG expressing RFP in THP-1 infected macrophages treated overnight with 50 µg/ml heparin two hours after internalization. Data from three independent experiments.

Heparin antimicrobial activity against Gram-positive bacteria has been long reported^26^, but its impact on the growth of *Mycobacterium* species has not been evaluated. When 7H9 medium was supplemented with 50 µg/ml of heparin, replication rates were not affected as measured by changes in absorbance patterns (OD_600_) (Fig. 2A). Since the effects could be exacerbated in a hostile environment such as within macrophages, increasing heparin concentrations (up to 250 µg/ml) in 7H9 medium also was assessed, but no changes in BCG growth were observed compared to untreated broth (Fig. 2B).

Heparin interacts with a myriad of serum proteins including complement factors^15^. During infection, heparin was added to complete RPMI medium with heat inactivated FBS. To evaluate if heparin is promoting Mtb killing through interaction with serum-proteins, 2.5 × 10^6^ bacilli were incubated in heparin containing C-RPMI for 72 hours in the absence of macrophages. Aliquots were spread onto 7H10 agar plates, and viable counts assessed. Again, heparin showed no direct bactericidal activity in C-RPMI medium (*p* = 0.216, Fig. S1B).

Altogether, these data demonstrate that heparin decreases Mtb replication through an indirect mechanism dependent on intracellular macrophage activity.

### Heparin treatment does not affect bacterial internalization

Heparin treatment has no impact on overall viable bacterial uptake compared to untreated control cells (Fig. 1A and C). However, the impact of heparin treatment on the percentage of host cells infected also was assessed. In these studies, heparin-treated macrophages were infected with red-fluorescent protein (RFP) labeled BCG and the percentage of infected cells was quantified by fluorescence microscopy two hours after internalization (Fig. 2D). In agreement with the total uptake results, heparin-treatment had no impact on the percentage of infected macrophages (*p* = 0.3) (Fig. 2C).

These data indicate that heparin’s impact on the intracellular mycobacterial burden is independent of its previously reported role in bacterial attachment and dissemination^11^.

### Heparin induces IL-1β secretion during Mtb infection

**Figure 3:**
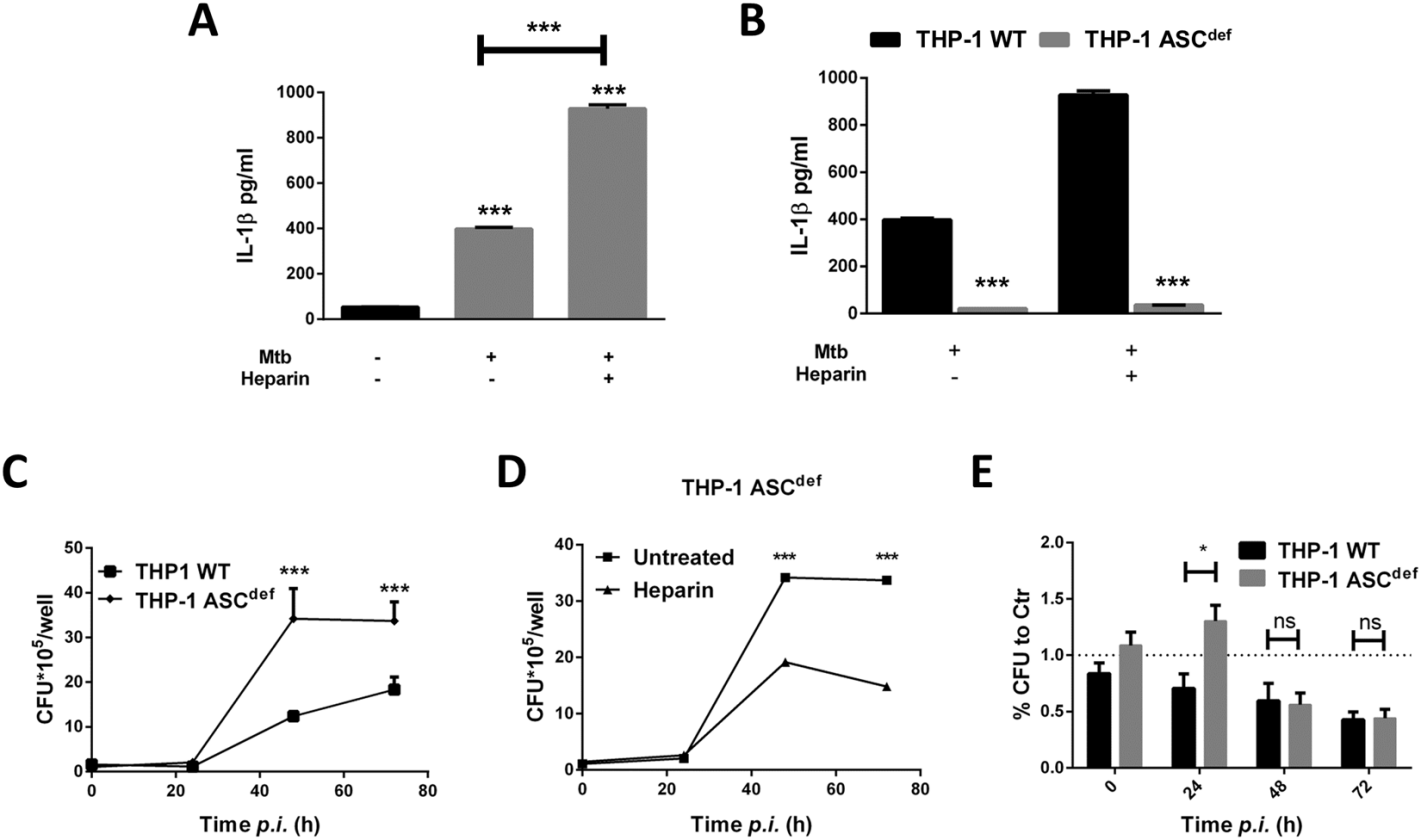
Heparin induces IL-1β secretion during Mtb infection in THP-1 macrophages. (**A**) IL-1β secretion in culture supernatants of heparin-treated macrophages 24 hours after Mtb infection. (**B**) IL-1β in ASC deficient macrophages 24 hours after Mtb infection. (**C**) Intracellular bacilli in Mtb infected ASC deficient and wild-type THP-1 macrophages. (**D**) Intracellular CFU in heparin-treated ASC deficient THP-1 macrophages. (**E**) Percentage of untreated control intracellular bacterial burden in heparin treated wild-type and ASC deficient THP-1 macrophages. For c and d macrophages were seeded in 48 well plates. Data from three independent experiments. *P < 0.05, **p < 0.01, ***p < 0.001.

IL-1β has been well correlated with a protective response to Mtb infection, thus the secretion of this cytokine was assessed in heparin-treated macrophages^5^. Heparin treatment alone does not induce IL-1β secretion in macrophages, although, 24 hours after Mtb or BCG infection, heparin-treated macrophages secrete significantly more IL-1β compared to untreated controls (mean difference was 38.38 ± 2.443 *pg*/ml, Fig. 3A). Infection with Mtb bacilli is known to induce IL-1β both through inflammasome- (NLRP3) dependent and independent pathways^27^. Thus, the contribution of heparin towards the induction of IL-1β was assessed after stimulation with NLRP3 specific ligands such as nigericin and ATP. Despite a marginally significant increase in IL-1β secretion by heparin-treated macrophages after LPS and nigericin treatment (mean difference was 12.79 ± 3.054 *pg*/ml, *p* = 0.014, Fig. S2A), Mtb infection generated a more robust response (mean difference was 529.90 ± 11.16*pg*/ml, Fig. 3A). In contrast, ATP-induced IL-1β is not affected by heparin treatment (*p* = 0.181, Fig. S2A), and heparin could not act as a sole first or second signal for inflammasome activation (Fig. S2B).

To confirm that heparin’s protective role during Mtb infection was dependent on IL-1β secretion, the impact of heparin treatment using caspase recruitment domain- (ASC)-deficient THP-1 (THP-1 ASC^def^) macrophages was assessed during Mtb infection. THP-1 ASC^def^ macrophages have impaired inflammasome activation and decreased IL-1β secretion after Mtb infection (Fig. 3B). In accordance with the previously reported protective effect of IL-1β, Δ_ASC_THP-1 macrophages show increased intracellular bacterial replication compared to parent THP-1 cells (*p* = 0.013, Fig. 3C). Surprisingly, after heparin treatment, THP-1 ASC^def^ macrophages still showed a decreased intracellular bacterial burden at 48 and 72 hours post infection compared to untreated cells. In fact, when normalized to the respective untreated controls (Fig. 3E), THP-1 ASC^def^ heparin-treated macrophages supported increased bacterial replication in relation to parent heparin-treated cells at 24 hours (*p* = 0.015), but by 48 and 72 hours post infection, intracellular bacterial replication was inhibited to similar levels in both cell lines (*p* = 0.93, Fig. 3E). These data suggest that heparin-induced IL-1β secretion is not responsible for the major differences in bacterial burden observed at later time points (48–72 hours) ion our infection model.

### Heparin inhibits hepcidin expression in THP-1 macrophages

Heparin is a known inhibitor of hepcidin expression in hepatocytes^22^ and hepcidin has been associated with increased replication of intracellular pathogens^28,29^. To test if heparin can inhibit hepcidin expression in macrophages, THP-1 macrophages were treated with 50 µg/ml heparin overnight and hepcidin mRNA expression was measured by qRT-PCR.

TLR4 activation induces hepcidin expression in macrophages^30,31^. To see if heparin could inhibit TLR-mediated hepcidin induction, heparin-treated macrophages were stimulated with LPS (500 ng/ml) for 24 hours and hepcidin gene transcription was assessed by qRT-PCR. These data were in accordance with a previous report that showed LPS treatment induces hepcidin expression in macrophages (13.61 ± 3.76 fold), however this response was not significantly affected by the addition of heparin (*p* = 0.3406, Fig. S3A).

We previously observed that iron supplementation greatly enhances TLR4-mediated hepcidin expression (unpublished data), thus the ability of heparin to inhibit hepcidin expression was assessed under these conditions (Fig. S3B). When grown in FeAC supplemented medium, LPS-stimulated macrophages showed a 39.78-fold (±3.53) induction in hepcidin mRNA levels compared to controls (*p* = <0.001); however, heparin-treated macrophages expressed significantly less hepcidin expression when stimulated under the same conditions (mean difference was 35.68 ± 3.56). In fact, LPS-mediated hepcidin induction is lower in heparin-treated macrophages grown in iron-supplemented versus iron-free media (Fig. S3B).

**Figure 4:**
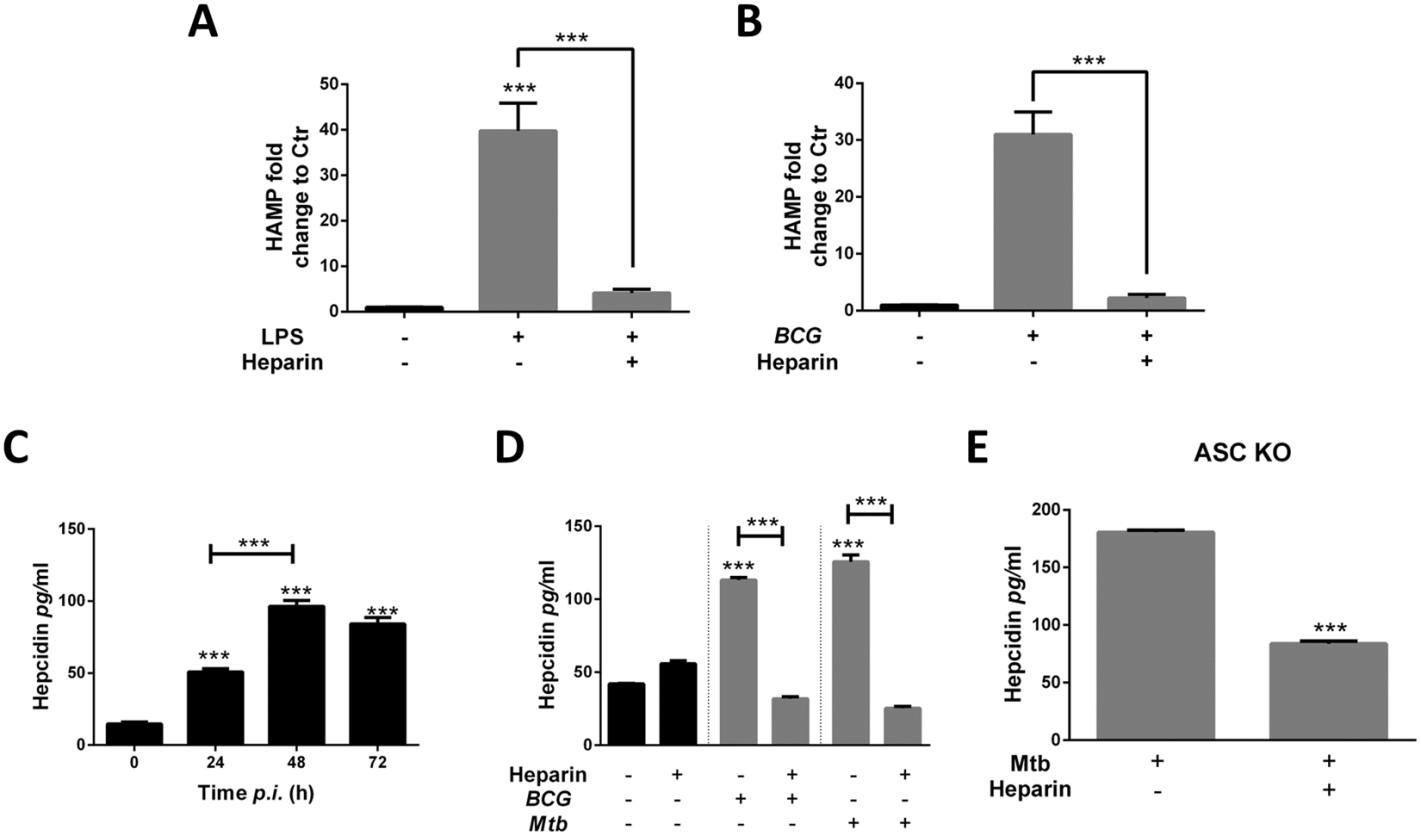
Heparin inhibits hepcidin expression in macrophages. (**A**,**B**) Hepcidin expression in heparin-treated macrophages 24 hours after LPS stimulation (**A**) or BCG infection (**B**) measured by qRT-PCR. (**C**) Hepcidin secretion in Mtb-infected THP-1 macrophages culture supernatants. (**D**) Hepcidin secretion by heparin-treated macrophages 48 hours after Mtb or BCG infection. (**E**) Hepcidin secretion by heparin treated ASC deficient THP-1 macrophages 48 hours after Mtb infection. Data from three independent experiments. *P < 0.05, **p < 0.01, ***p < 0.001.

BCG and Mtb activate TLR4 signaling, and have been shown to induce hepcidin in iron-supplemented medium (unpublished data). Thus, the inhibition of BCG-induced hepcidin expression in THP-1 macrophages also was assessed. After BCG infection, heparin-treated macrophages showed decreased hepcidin mRNA levels compared to untreated controls (mean difference was 28.72 ± 2.32 fold, Figs 4A,B and S5). Consistently, hepcidin secretion is decreased four-fold in the culture supernatants from heparin-treated macrophages after BCG (Fig. 4D) and Mtb infection (Fig. 4D,E).

Overall, these results show that heparin inhibits Mtb-induced hepcidin expression in macrophages.

### Heparin inhibits hepcidin-mediated ferroportin internalization and degradation

**Figure 5:**
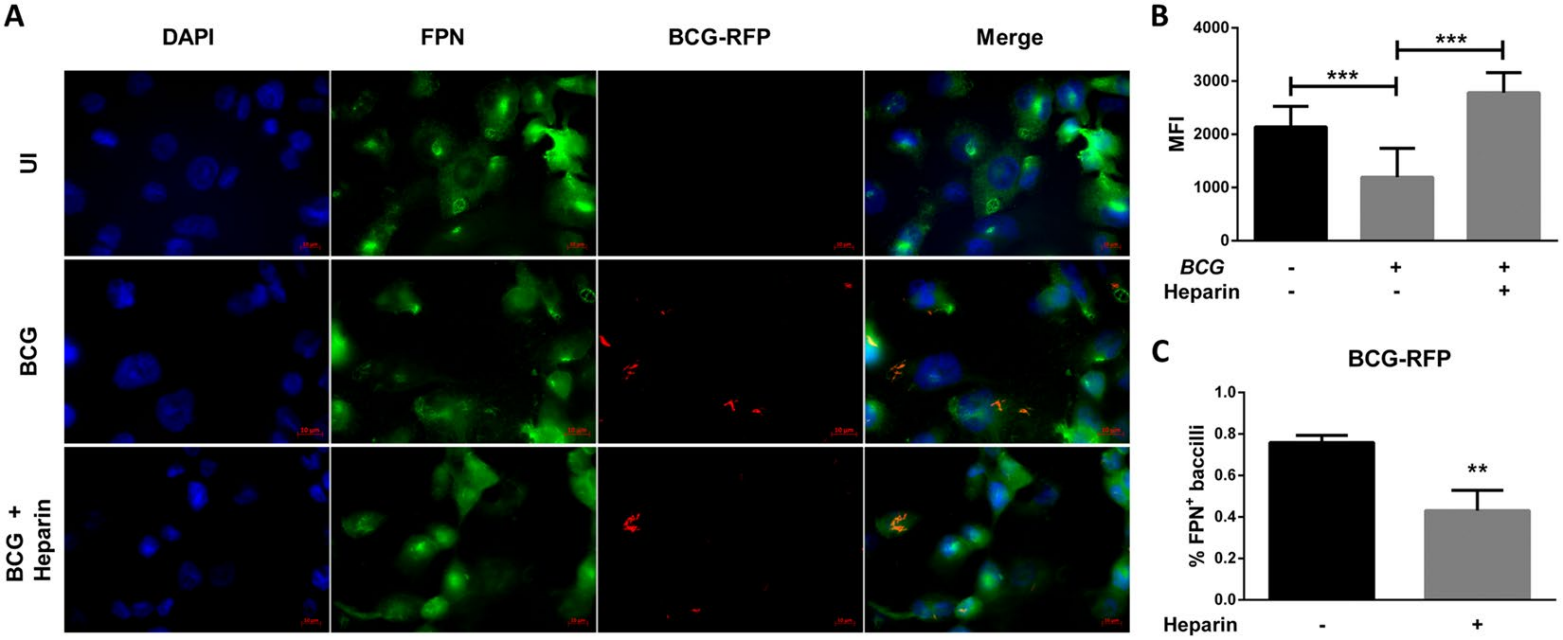
Heparin-treated macrophages have increased ferroportin levels. (**A**) Ferroportin expression in heparin-treated macrophages 48 hours after BCG infection (63X). (**B**) Ferroportin expression in heparin-treated macrophages 48 hours after BCG infection, measured as Pixel MFI/cell from a minimum of 20 cells in three different fields (40X) from three independent experiments. (**C**) Percentage of BCG bacilli colocalized with ferroportin-FITC staining (overlapping red and green pixels). Data from three independent experiments. **p < 0.01, ***p < 0.001.

Secreted hepcidin binds to the iron exporter protein ferroportin, leading to its internalization and degradation^32^. Heparin can prevent LPS-mediated hepcidin expression and consequent ferroportin internalization in macrophages (Fig. S4). Like LPS treatment, BCG infection leads to decreased surface ferroportin levels 48 hours after infection, which overlaps with maximum differences in hepcidin expression (Fig. 5A). Quantification of mean pixel fluorescence intensity shows that infected cells express 44% less ferroportin and 16 times more hepcidin than uninfected controls (Fig. 5B). Interestingly, 48 hours after infection, 75.8 ± 0.02% of intracellular BCG bacilli in macrophages would co-localize with ferroportin, although in heparin-treated macrophages only 43 ± 0.5% of BCG bacilli overlap with ferroportin staining (Figs 5C and S6). This shows that heparin inhibits hepcidin-mediated ferroportin internalization and degradation.

### Heparin decreases intracellular iron levels

**Figure 6:**
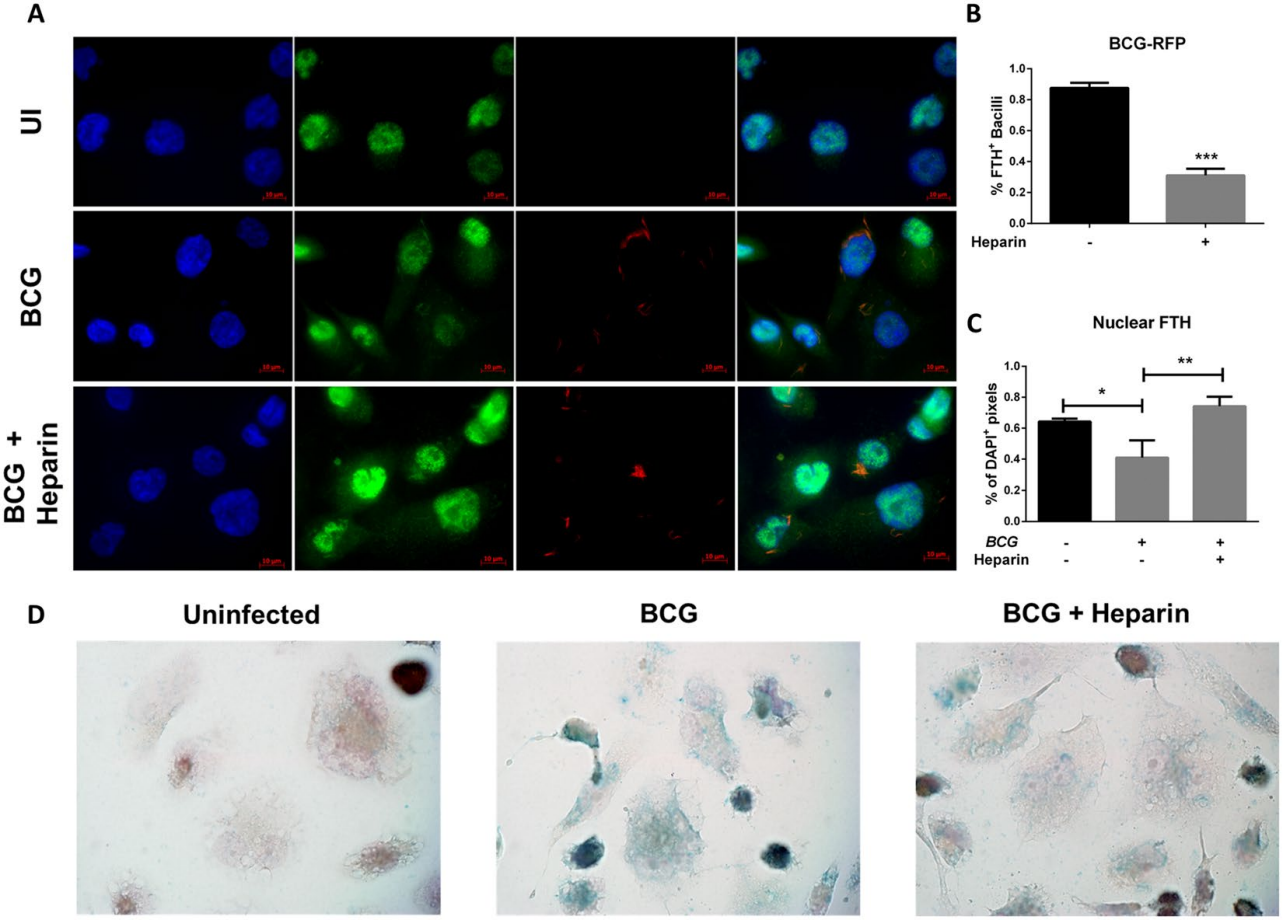
Heparin decreases iron availability to intracellular mycobacterial bacilli. (**A**) Ferritin expression in heparin-treated macrophages 48 hours after BCG infection. (**B**) Percentage of ferritin -positive BCG bacilli in five fields from three independent experiments as represented in (**A**). (**C**) Nuclear ferritin in heparin-treated macrophages 48 hours after BCG infection measured by percent of double-positive pixels (FITC and DAPI). (**D**) Total intracellular iron staining (Prussian blue) in heparin-treated macrophages 48 hours after BCG infection. Data from three independent experiments. *p < 0.05, **p < 0.01, ***p < 0.001.

Increased intracellular iron is normally associated with increased ferritin expression. Similarly, increased hepcidin secretion and decreased ferroportin expression are associated with increased intracellular iron sequestration. Macrophages infected with BCG express higher ferritin levels than uninfected controls, also suggesting increased intracellular iron sequestration (Figs 6A and S7A and B). Heparin treatment can slightly decrease ferritin expression after BCG infection but not to levels that resemble uninfected cells (Figs 6A and S7A and B).

*Mycobacterium tuberculosis* and BCG can sequester iron from cytoplasmic iron storage compartments of infected macrophages. In this study, intracellular ferritin distribution was assessed after BCG infection. Consistent with western blot data, BCG-infected macrophages have higher levels of ferritin compared to uninfected controls resulting from increased intracellular iron sequestration (Fig. 6A). Surprisingly, in uninfected macrophages ferritin localizes to the nucleus, with very little distribution in the cytoplasm. Upon BCG infection, ferritin is found in the cytoplasm (Fig. 6C). This can be confirmed microscopically by strong association between infecting bacilli and labeled ferritin (87.6 ± 1.7% ferritin-positive intracellular bacilli, Fig. 6B). Heparin has no impact on intracellular ferritin levels in BCG infected macrophages, but it seems to alter its intracellular distribution with increased nuclear localization (Fig. 6C) and decreased association with the BCG bacilli (31.1 ± 2.1%, Fig. 6B).

Decreased ferritin without increased iron export leads to increased free iron levels which have a complex role in bacterial survival and replication. Free cytoplasmic iron is easily accessible by the intracellular bacteria, but if the iron is transported to the phagolysosome, it contributes to reactive oxygen species production through the Fenton reaction which strongly promotes bacterial killing. To evaluate the impact of heparin treatment on the intracellular labile iron pool (LIP), the percentage of calcein fluorescence quenching in heparin-treated macrophages was assessed after LPS stimulation. LPS treatment mimics some aspects of BCG or Mtb infection with strong hepcidin and ferritin induction, ferroportin down regulation and increased intracellular iron levels (Fig. S7D). Iron supplementation promotes a moderate but significant increase in the intracellular LIP, however, this is not altered by LPS stimulation until six hours after treatment. After 24 or 48 hours post treatment, LPS-treated cells have much higher intracellular LIP levels, as observed by increased calcein-quenching (67.1% decrease in fluorescence). Nonetheless, LPS-induced LIP is not changed in heparin-treated macrophages suggesting that heparin does not alter intracellular LIP levels. Pam3CSK4, the TLR2/TLR1 activator, induces intracellular iron arrest by direct transcriptional down regulation of ferroportin and through a hepcidin-independent mechanism (unpublished data). Pam3CSK4-treated macrophages show similar intracellular LIP with LPS-treated cells, suggesting regulation of LIP in macrophages is independent of hepcidin expression.

Ferritin expression is strongly associated with iron storage levels, but does not represent total iron content of the cell; total intracellular iron levels can be assessed by Prussian blue (PB) staining. Consistent with our previous observations, uninfected macrophages have low iron content, as seen by low staining (Fig. 6D). However, upon BCG-infection, intracellular iron content is increased (12.7 ± 5.1%). In contrast, heparin-treated infected macrophages show significantly decreased intracellular iron levels compared to uninfected controls (*p* = 0.01) and untreated infected cells (*p* = 0.007) (Figs 6D and S7C). Overall, these results demonstrate that heparin decreases iron availability to intracellular mycobacteria in macrophages.

### Heparin can only inhibit intracellular BCG and Mtb replication under high intracellular iron conditions

**Figure 7:**
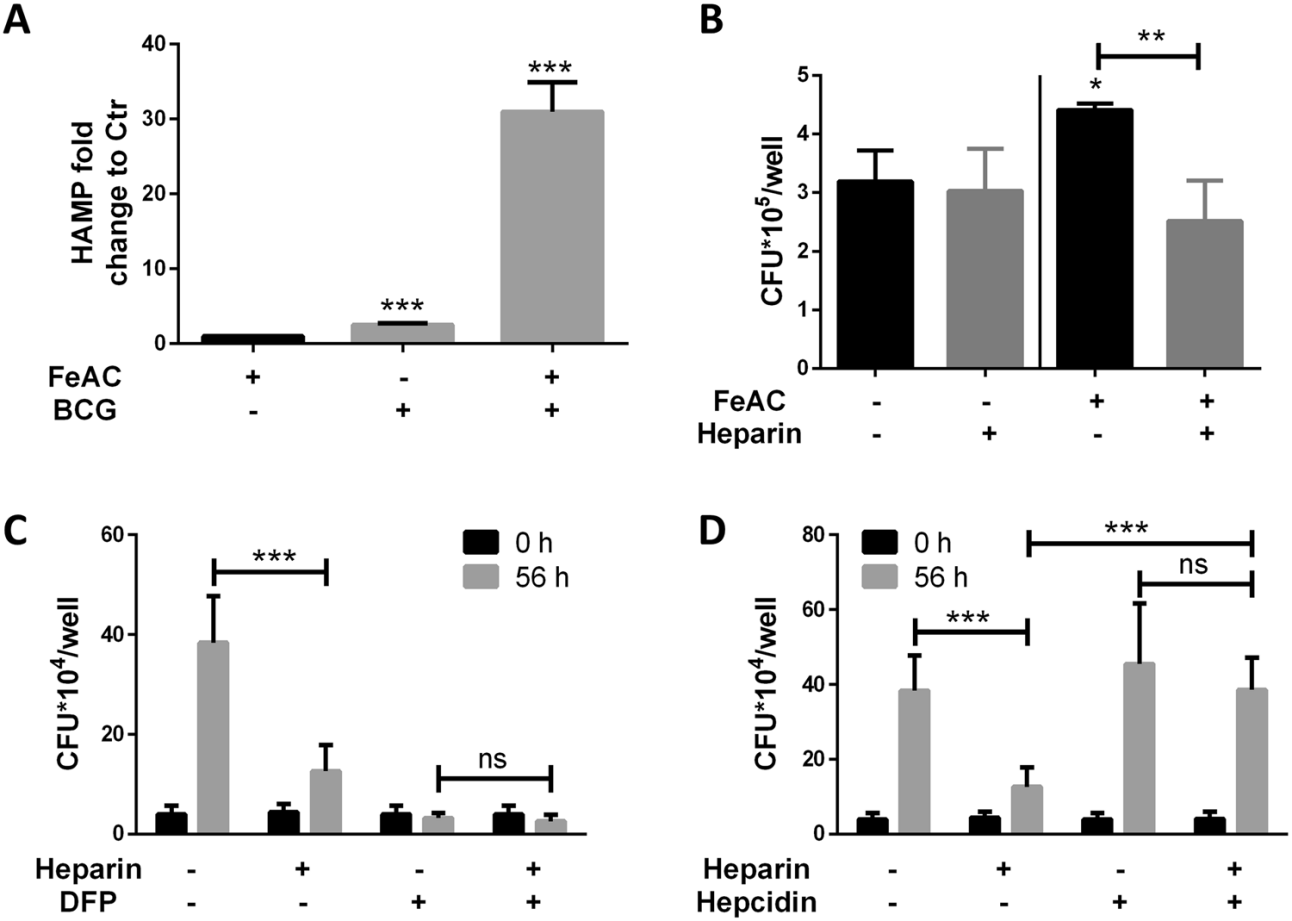
Hepcidin rescues intracellular Mtb replication in heparin-treated macrophages. (**A**) Hepcidin mRNA expression in BCG-infected macrophages with and without iron supplementation. (**B**) Intracellular bacterial burden in heparin-treated macrophages with and without iron supplementation. (**C**) Intracellular bacterial burden in heparin-treated macrophages in presence of iron chelator deferiprone at 0 and 56 hours post infection. (**D**) Intracellular bacterial burden in heparin-treated macrophages supplemented with hepcidin synthetic peptide. For b macrophages were seeded in 48 well plates. For c and d macrophages were seeded in 96 well plates. Data from three independent experiments. *p < 0.05, **p < 0.01, ***p < 0.001.

*Mycobacterium bovis* BCG replication is generally well contained in human macrophages, although virulence can be promoted by different mechanisms^33^. Iron supplementation promotes enhanced BCG replication in macrophages which can be inhibited by heparin treatment (Fig. 7B). Furthermore, it has been shown previously that BCG can only induce hepcidin expression in macrophages when grown in iron-supplemented medium (Fig. 7A), thus, the impact of heparin treatment was assessed in non-iron supplemented medium. When compared to untreated controls, heparin treatment had no impact on intracellular bacterial replication in plain RPMI (no iron added) (Fig. 7B), further connecting heparin with hepcidin down regulation and decreased iron availability.

Deferiprone is an iron chelator clinically approved for the treatment of iron overload disorders and thalassemia^34–36^. During Mtb infection, treatment with deferiprone significantly decreases intracellular bacterial replication in macrophages (Fig. 7C). In agreement with heparin’s impact in iron availability to intracellular bacilli, heparin/deferiprone -treated macrophages have similar bacterial burdens to deferiprone -alone treated controls (Fig. 7C).

### Hepcidin supplementation increases intracellular bacilli replication in heparin-treated macrophages

Hepcidin_25_ synthetic peptide is commercially available and has been shown to maintain the iron modulatory function of the native protein^37^. To prove that heparin-mediated hepcidin inhibition is responsible for decreased intracellular bacterial burden at later time points of infection, heparin-treated macrophages were supplemented with hepcidin_25_ synthetic peptide. As predicted, hepcidin supplementation rescued intracellular bacterial replication in heparin-treated macrophages; however, no significant increase in untreated macrophages was detected (Fig. 7D).

## Discussion

Notwithstanding the efforts to eradicate it, tuberculosis has again become the leading cause of death due to an infectious disease^38,39^. The increase in infections with multidrug-resistant and extensively-drug resistant strains makes the use of therapeutics as our only effective intervention strategy a unsustainable plan. Thus, a truly effective control strategy requires that new therapeutics and a more effective vaccines are developed^39^. In this report a protective immunomodulatory role for heparin during Mtb infection in macrophages is described. We demonstrate that heparin modulates macrophage iron status, and decreases iron availability for intracellular bacilli, thus limiting bacterial replication.

*Mycobacterium tuberculosis* bacilli persist and reside inside alveolar macrophages. To replicate within the phagosome of these cells, Mtb bacilli must recruit essential nutrients, such as iron to this vesicular compartment^40,41^. Despite the important role of macrophages in iron recycling from erythrophagocytosis, free iron is extremely scarce inside the macrophage being rapidly redistributed extracellularly through the iron exporter ferroportin or sequestered by the iron storage protein ferritin.

*Mycobacterium tuberculosis* bacilli possess a myriad of mechanisms for iron scavenging inside the host. For example, PAMPs activate TLR2 and TLR4 signaling and promote intracellular iron sequestration in macrophages through induction of hepcidin and down regulation of ferroportin (unpublished data). In addition, increased expression of mycobactin and carboxymycobactin siderophores efficiently recruit and scavenge iron for use by the intracellular mycobacteria within the phagosome^42–44^. Carboxymycobactin is the major iron-chelator for both free and protein-bound iron in the macrophage phagosome and cytoplasm^44–46^, while surface mycobactin acts as a membrane chelator and iron-transporter recovering iron from carboxymycobactin and host ferritin. Both molecules are essential for iron acquisition and pathogenesis as shown by the severe attenuation of Mtb knock-out strains with impaired siderophore synthesis^47^.

In this study, heparin treatment significantly inhibited Mtb-mediated hepcidin secretion (Fig. 4) culminating in increased ferroportin expression 48 hours after infection (Fig. 5). Poli *et al*. have previously shown that heparin can inhibit hepcidin expression in hepatocytes, but it is shown here that this highly sulfated glycosaminoglycan has a similar impact in TRL-mediated hepcidin expression in myeloid cells.

*Mycobacterium tuberculosis* infection promotes increased intracellular iron sequestration and ferritin expression (Fig. 6). Luo *et al*.^42^ showed that *Mycobacterium paratuberculosis* mycobactin J (mbtJ) rapidly disperses from the phagosome in host lipid cellular components, accessing the macrophage intracellular iron pool. Iron loaded mbtJ localizes with lipid droplets that are later associated with the phagosome. Here, heparin had no impact in the intracellular labile iron pool (Fig. S7), but greatly decreased total intracellular iron levels, potentially protein-bound (Fig. 6D). In macrophages, ferritin mostly localizes to the nucleus with minimal cytoplasmic distribution (Fig. 6). Ferritin nuclear translocation has been previously reported in murine macrophages during iron overload^48^, but its mechanism and function remain unclear. Macrophages infected with BCG bacilli have decreased ferritin nuclear co-localization, despite the increase in ferritin expression (Fig. 6A), while heparin-treated macrophages show a nuclear ferritin distribution comparable to uninfected cells. Interestingly, heparin also decreases ferritin -BCG co-localization further decreasing iron availability for the intracellular bacilli (Fig. 6).

Decreased intracellular iron availability through chelation therapy significantly limits intracellular mycobacterial growth^6,49^ and Fig. 7). In our study, heparin impacts intracellular BCG replication in the presence of iron and can be counteracted by the addition of hepcidin to the medium (Fig. 7), indicating that heparin-mediated hepcidin inhibition and the decrease in intracellular iron availability are the major action mechanisms limiting intracellular replication. *De facto*, a similar mechanism has been described in IFNγ-activated murine macrophages^50,51^. In that report, IFNγ-induced ferroportin expression contributed to efficient control of *Salmonella enterica* intracellular replication^51^.

Alveolar macrophages and potentially type II pneumocytes are the primary cell targets during Mtb infection^52^. Heparin is known to prevent mycobacterial attachment to and internalization of type II pneumocytes^11,12^. However, macrophages actively phagocytose opsonized bacteria through Fcγ receptors and the complement receptor. In this study, heparin treatment of macrophages had no impact on bacterial attachment and internalization, but intracellular replication was reduced at later time points compared to untreated controls. Phagocytosis of opsonized Mtb bacilli by resident macrophages in the lung leads to phagosome acidification and lysosome fusion, increased reactive oxygen and nitrogen species, and recruitment of antibacterial peptides culminating in bacterial clearance^53^. Nonetheless, Mtb bacilli activate an arsenal of virulence factors which block efficient macrophage antibacterial functions^53,54^. An example of these Mtb blocking factors include secretion of phosphotyrosine protein phosphatase (Ptpa) immediately upon Mtb macrophage internalization. This protein inhibits host membrane fusion proteins and host V-ATPases required for phagosome maturation and acidification. An additional factor is Mtb nucleoside diphosphate kinase (NdK) which inhibits phagolysosome fusion and NOX2-mediated ROS production^55,56^. It would be interesting to determine if heparin can counteract bacterial factors and impact early events after phagocytosis such as phagosome maturation, lysosome fusion and promote efficient bacterial killing.

The use of porcine unfractionated heparin (UFH) raises limitations to direct translation from our study into a novel therapeutic strategy. UFH is a mixture of heparins of variable sizes with limited bioavailability and extremely variable anticoagulant pharmacological properties^10^. Still, over the last decade extensive efforts have been made to develop modified heparins with improved and more targeted pharmacological activities. Recently, Poli *et al*.^25^ revealed that glycol-split non-anticoagulant heparin fractions can mimic intracellular signaling of UFH leading to hepcidin inhibition in hepatocytes. Future studies will examine if these non-anticoagulant heparins can prevent Mtb-mediated iron sequestration in macrophages and limit intracellular bacterial replication while limiting side effects.

Iron dysregulation has been long associated with increased risk of developing TB^57,58^. Moreover, hepcidin serum levels are strongly correlated with *Mtb*– HIV co-infection^59–61^. Hepcidin expression and decreased iron export have been shown to increase HIV replication rates in macrophages^6^, reinforcing the importance of this hormone in co-infection. This study leads the way towards a potential use of hepcidin inhibitors such as heparin, as an efficient therapeutic strategy against TB, and a promising prospect for immunomodulatory therapies in HIV-Mtb co-infected patients.

## Methods

### Cell culture and macrophage differentiation

The THP-1 monocytic cell line was obtained from ATCC (TIB-202), maintained in complete RPMI with 2 mM glutamine and supplemented with 10% fetal bovine serum (C-RPMI). For differentiation into a macrophage-like phenotype, 8 × 10^5^ cells/ml were treated with 50 nM phorbol 12-mytistate 13-acetate for 24 hours and rested overnight in C-RPMI with 100 µM ferric ammonium citrate (FeAC). When stated, 50 µg/ml heparin (≈10U/ml) was subsequently added to the medium during overnight resting. THP-1 cells deficient in PYD, the CARD Domain Containing (THP-1 ASC^def^) cells and the parent strain, all were obtained from Invivogen (CA USA), and maintained and differentiated as described above for the ATCC THP-1 original cell line.

### Bacterial strains and infection

The strains used in this study were *Mycobacterium bovis* BCG Pasteur, and Mtb Erdman generously provided by Dr Jeff. Cox (UC Berkley, CA USA). Strain BCG Pasteur expressing RFP was generously provided by Dr. Andrew Mellor (Augusta University, GA, USA). Bacteria were grown to an OD_600_ ≈ 0.8 in 7H9 medium supplemented with ADC, 5% glycerol and 0.5% Tween 80, and aliquots frozen at −80 °C. Frozen aliquots were thawed, serially diluted and plated on 7H10 agar medium containing 10% ADC for three weeks at 37 °C. Viability was measured as colony forming units/ml (CFU/ml). Prior to infection, BCG or Mtb bacilli were passed through a 21G tuberculin syringe and opsonized for 2 hours in RPMI with 10% non-heat inactivated horse serum at 37 °C with gentle rocking.

For infection, 1.5 × 10^6^, 3 × 10^5^ or 8*10^4^ PMA-differentiated THP-1 macrophages were incubated in C-RPMI with opsonized bacteria in 12, 48 or 96 well plates respectively at a multiplicity of infection of five to 10 bacilli per host cell for two hours at 37 °C with 5% CO_2_. After internalization, macrophages were washed three times with warm PBS. After washing, C-RPMI containing 50 µg/ml gentamicin and 50 µg/ml heparin was added to the infected cells and maintained throughout the experiment. For intracellular bacterial burden quantification, cells were lysed at indicated time points with 0.1% TritonX-100 for 10 minutes and serial dilutions plated on 7H10 agar medium containing 10% ADC. CFUs were counted twice after incubation for 19 to 23 days at 37 °C.

### Heparin bacteriostatic and bactericidal assay

BCG bacilli were grown in complete 7H9 medium with increasing heparin concentrations (50 to 250 µg/ml), in T25 flasks, at a starting OD_600_ ≈ 0.001. Growth was measured daily by changes in OD_600_ of 100 µl aliquots in 96 well flat bottom plates and assayed using *Powerwave* XS2 (Biotek, VT USA).

Mtb Erdman bacilli were grown similarly to BCG, but changes in absorbance were measure in 13 mm diameter spec tubes and assayed using a *Spectronic 20* + spectrophotometer.

To assess heparin bactericidal activity, 2.5 × 10^6^ bacteria were incubated in C-RPMI with 50 µg/ml heparin for 72 hours at 37 °C with 5% CO_2_, and serial dilutions plated on 7H10 agar medium containing 10% ADC. CFUs were counted twice after incubation for 19 to 23 days at 37 °C.

### RNA extraction and real-time PCR

Total cellular RNA from 1 × 10^6^ THP-1 macrophages was extracted using TRIzol (Invitrogen, Thermo Fisher Scientific, MA USA) following the manufacturer’s protocol and reverse transcribed into cDNA using SuperscriptIII First strand cDNA synthesis Kit (Invitrogen) with poly dT20 primers. Quantitative PCR (qPCR) was performed using Bio- Rad IQ SYBR green supermix (Bio-Rad, CA USA) in a iQ™5 Real-Time PCR Detection System. All values were normalized against GAPDH (ΔCT = CT [HAMP] − CT [GAPDH]). Fold change in expression was calculated as 2^−ΔΔCT^, where ΔΔCT = ΔCT (test sample) − ΔCT (control). The primer sequences for the genes examined were the following: human Hamp, forward, 5=-GGATGCCCATGTTCCAGAG-3=; reverse, 5=-AGCACATCCCACACTTTGAT-3=; human GAPDH, forward, 5=-GCCCTCAACGACCACTTTGT -3=; reverse, 5=-TGGTGGTCCAGGGGTCTTAC-3=.

### Hepcidin secretion quantification

Hepcidin levels in culture supernatants were determined using the human hepcidin DuoSet Elisa Kit (R&D Systems, MN USA), per manufacturer’s recommendations.

### Protein extraction and Western blot analysis

For western blot, 1 × 10^6^ cells were cultured in 6-well plates, washed twice with ice-cold PBS and lysed with ice-cold IP lysis for 30 minutes on ice. Cell lysates were further disrupted manually by vigorous pipetting and vortexing. After centrifugation (10,000 × g) for 15 minutes at 4 °C, supernatants were collected and stored at −20 °C until analyzed.

Total protein content was determined by using the BCA protein estimation assay kit (Pierce, Thermo Fisher Scientific MA USA). Samples (20 µg) were mixed with Laemmli buffer (1x final concentration), heated at 70 °C for 10 minutes, and proteins were electrophoretically separated on a 15% sodium dodecyl sulfate (SDS)–polyacrylamide gel. The proteins were transferred to a PVDF membrane (Bio-RAD), which was then pre-incubated with blocking solution (5% nonfat dry milk in Tris-buffered saline containing 0.01% Tween20 [TBST], pH 7.4) for one hour, followed by overnight incubation with 1 μg of anti-FTH1 (ferritin) rabbit monoclonal antibody (Cell signaling Tech,, MA USA) and 1 µg Anti-GAPDH rabbit monoclonal antibody (cell signaling) at 4 °C. After primary incubation, the membrane was washed 3x with TBST and incubated for one hour with secondary anti-rabbit HRP conjugated antibody (Cell signaling Tech).

All incubations and wash steps were performed at room temperature except when otherwise stated. Cross-reactivity was visualized by using enhanced chemiluminescence (SuperSignalWestPico; Pierce).

### Immunofluorescence microscopy

Anti-ferroportin and anti-hepcidin antibodies for ferroportin and hepcidin detection were kindly provided by Dr. Tara Arvedson, and immunofluorescence staining was done as previously described.

Briefly, 2 × 10^5^ THP-1 macrophages were grown and differentiated in eight or 16 well chamber microscopy slides and infected as described above, fixed with 4% PFA overnight and permeabilized with 0.1% Triton X-100. For ferroportin staining, cells were incubated with 2 μg/ml mouse antibody diluted in C-RPMI overnight. For detection, cells were incubated with 2 µg/ml goat anti-mouse alexa-fluor-488 (Invitrogen, Thermo Fisher Scientific. MA USA) at 4 °C for two hours. Cells were counterstained with DAPI.

For hepcidin staining, cells were treated with infected, fixed and permeabilized as described above, and stained with 2 μg/ml mouse anti-hepcidin antibody overnight at 4 °C.

Slides were imaged in a Zeiss Axiovert 200 M microscope at 40X and 63X and images acquired with Axiocam MRm grey scale camera.

### Prussian blue for iron staining

Four-hundred thousand THP-1 macrophages were grown and differentiated in eight well chamber microscopy slides as described above. After infection, cells were fixed with 4% formaldehyde in PBS for 10 minutes at room temperature, washed with PBS and stained twice with 4% HCl and 4% K4[Fe(CN)6] · 3H2O (1:1v/v) solution for 25 minutes (Prussian blue stain Kit Polysciences, Warrington, PA USA). After washing with PBS, cells were counterstained with filtered 1% Nuclear Fast red solution for five to 10 minutes. After gentle washing with PBS and double-distilled H_2_O, slides were mounted and imaged on an Axiovert 40CFL microscope. Images were acquired on a Axiocam MRC5 color camera with 20X and 40X lenses.

### Intracellular labile iron pool (LIP) quantification

Intracellular LIP was measured through a calcein quenching assay as previously described and adapted to flow cytometry analysis. Briefly, 3 × 10^5^ THP-1 macrophages were seeded in 48-well plates and treated with LPS or Pam3CSK4 (synthetic triacylated lipopeptide that activates the TLR2/TLR1 heterodimer) up to 48 hours in iron supplemented medium. At each timepoint, cells were washed twice with warm PBS, stained with calcein-AM (Invitrogen) for 15 minutes at room temperature, washed again with warm PBS, trypsininzed, resuspended in FACS buffer and analyzed by flow cytometry before and after iron chelation with deferiprone (DFP). Quenched fluorescence was determined as percentage of Mean Fluorescence Intensity before iron chelation (xMFI) to 10 minutes after addition of DFP (xMFI_DFP_) $(\frac{{xMFI}}{({{xMFI}}_{{DFP}})}\times 100)$. Cells grown in non-iron supplemented medium were used as negative controls.

### Image analysis

Image analysis and mean pixel fluorescence intensity were determined with Zeiss Axiovision Rel 4.8.1 software. Colocalization and Prussian blue staining were quantified with imageJ 1.51 K software. Grey scale images were threshold for background and converted to binary files for automatic shape analysis. Bacilli-protein colocalization was determined as shapes with double positive pixels. Protein-protein colocalization was determined by double positive pixel areas.

Prussian blue staining was quantified in 200x color image thresholds for background and determined as percentage of blue pixel area over total pixel area averaged from at least four different fields from three independent experiments.

### Statistics

All data are presented as means ± SD. Statistical significance differences between two groups were determined using Student’s t test or 2-way ANOVA (Bonferroni) for multiple group comparison with GraphPad Prism software (CA, USA).

## Electronic supplementary material

Supplementary information
