# Supplementary material for: Heparin inhibits intracellular Mycobacterium tuberculosis bacterial replication by reducing iron levels in human macrophages
Source: Sci Rep. 2018 May 8;8:7296. doi: 10.1038/s41598-018-25480-y (PMC5940867; doi:10.1038/s41598-018-25480-y)
Supplement: Supplementary file 1 — Supplementary information [file 41598_2018_25480_MOESM1_ESM.pdf]

Heparin inhibits intracellular *Mycobacterium tuberculosis* bacterial replication by reducing iron levels in human macrophages.

Rodrigo Abreu*, Lauren Essler*+, Allyson Loy*+, Frederick Quinn*, and Pramod Giri*[1]

*** Department of Infectious Diseases, College of Veterinary Medicine, University of Georgia, Athens GA, 30602 USA**

+ Department of Microbiology, University of Georgia, Athens GA, 30602 USA

[1] Corresponding author - Pramod K Giri, **Department of Infectious Diseases, College of Veterinary Medicine, University of Georgia,** 501 D.W Brooks Drive, Athens GA, 30602, pgiri@uga.edu, 706-542-2548

Supplementary information

Fig S1: **Heparin has no direct impact on host cell or bacteria viability**. a) THP-1 cell viability after heparin (50µg/ml) treatment relative to untreated control. b) Viability of Mtb bacilli after 72 hours treatment with heparin (50µg/ml) in C-RPMI without macrophages. Data from two independent experiments.

Fig S2: **Heparin induces NLRP3-mediated IL-1β secretion by macrophages.** a) IL-1β secretion in culture supernatants of macrophages primed with LPS overnight and treated with nigericin or ATP for inflammasome activation, with or without heparin. b) IL-1β secretion in culture supernatants of macrophages primed with LPS and treated with heparin or nigericin for inflammasome activation. Data from three independent experiments. *$P<0.05$, ***$p<0.001$

Fig S3: **Heparin inhibits LPS-mediated hepcidin expression in iron supplemented media.** Hepcidin expression in heparin-treated macrophages 24 hours after LPS stimulation in normal RPMI media (a) or iron supplemented media (b) measured by qRT-PCR. Data from three independent experiments. ***$p<0.001$

Fig S4: **Ferroportin surface expression in THP1 macrophages treated with LPS in heparin supplemented media.** THP-1 macrophages differentiated and treated with LPS as described in the material and methods, stained for surface ferroportin and analyzed by flow cytometry.

Fig S5: **Heparin inhibits BCG-induced hepcidin expression.** a) Hepcidin expression in heparin-treated macrophages 48 hours after BCG infection (63X). b) Pixel MFI/cell from a minimum of 20 cells in three different fields represented in A from three independent experiments.

Fig S6**: BCG and ferroportin colocalization.** Ferroportin expression in RFP-BCG infected macrophages (100X). THP-1 macrophages differentiated and infected with RFP-BCG as described the material and methods and stained for ferroportin.

Fig S7: **Mycobacterial infection has no impact on the intracellular labile iron pool of macrophages.** a) Ferritin (left) and loading control β-actin (right) expression in heparin-treated THP-1 macrophages 48 hours after BCG infection. b) Relative Ferritin bands (upper FTH^1, lower FTH^2) intensity normalized to loading control as shown in A. c) Percentage of Prussian blue pixels area to total stained pixel surface area in heparin-treated macrophages 48 hours after BCG infection. d) Labile iron levels in heparin-treated macrophages three and 48 hours after LPS or Pam3CSK4 stimulation. A and B data is representative of three independent experiments. C and D data from three independent experiments. *$p<0.05$, **$p<0.01$, ***$p<0.001$

Fig S1

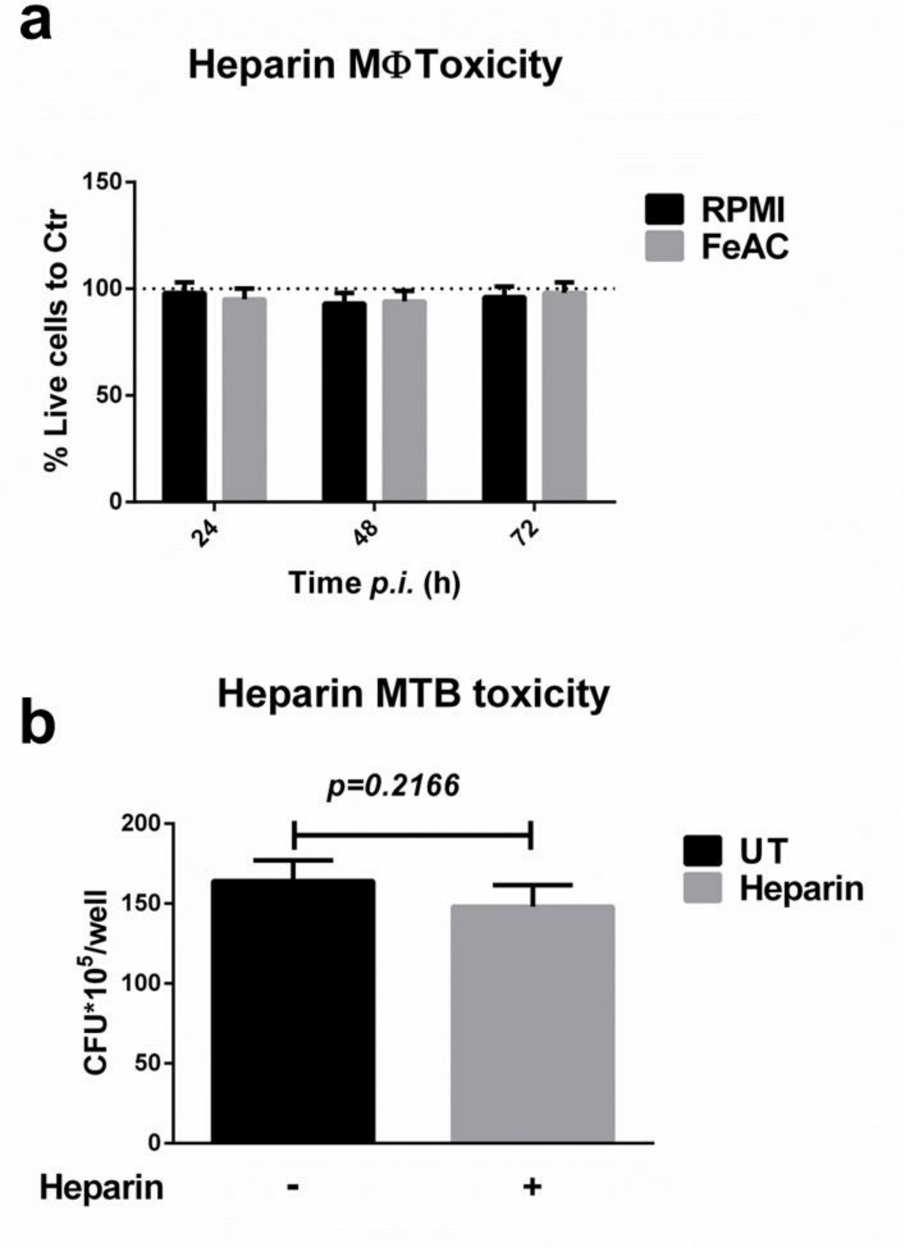
a
Heparin MΦToxicity
% Live cells to Ctr
150
100
50
0
24
48
72
Time p.i. (h)
RPMI
FeAC
b
Heparin MTB toxicity
p=0.2166
CFU*10^5/well
200
150
100
50
0
Heparin
-
+
UT
Heparin

Fig S2

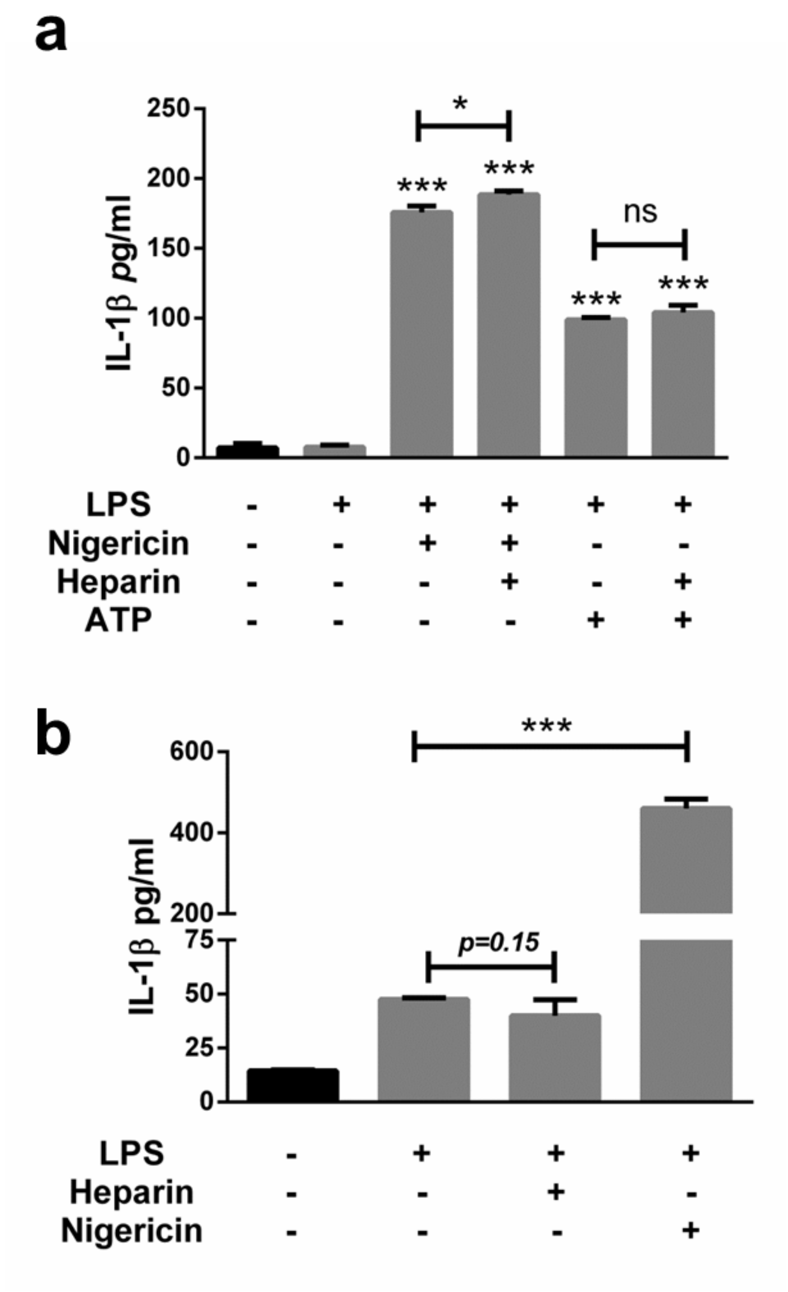

a
IL-1β pg/ml
250
200
150
100
50
0
*
ns
***
***
***
***
LPS - + + + + +
Nigericin - - + + - -
Heparin - - - + - +
ATP - - - - + +
b
IL-1β pg/ml
600
400
200
75
50
25
0
***
p=0.15
LPS - + + +
Heparin - - + -
Nigericin - - - +

Fig S3

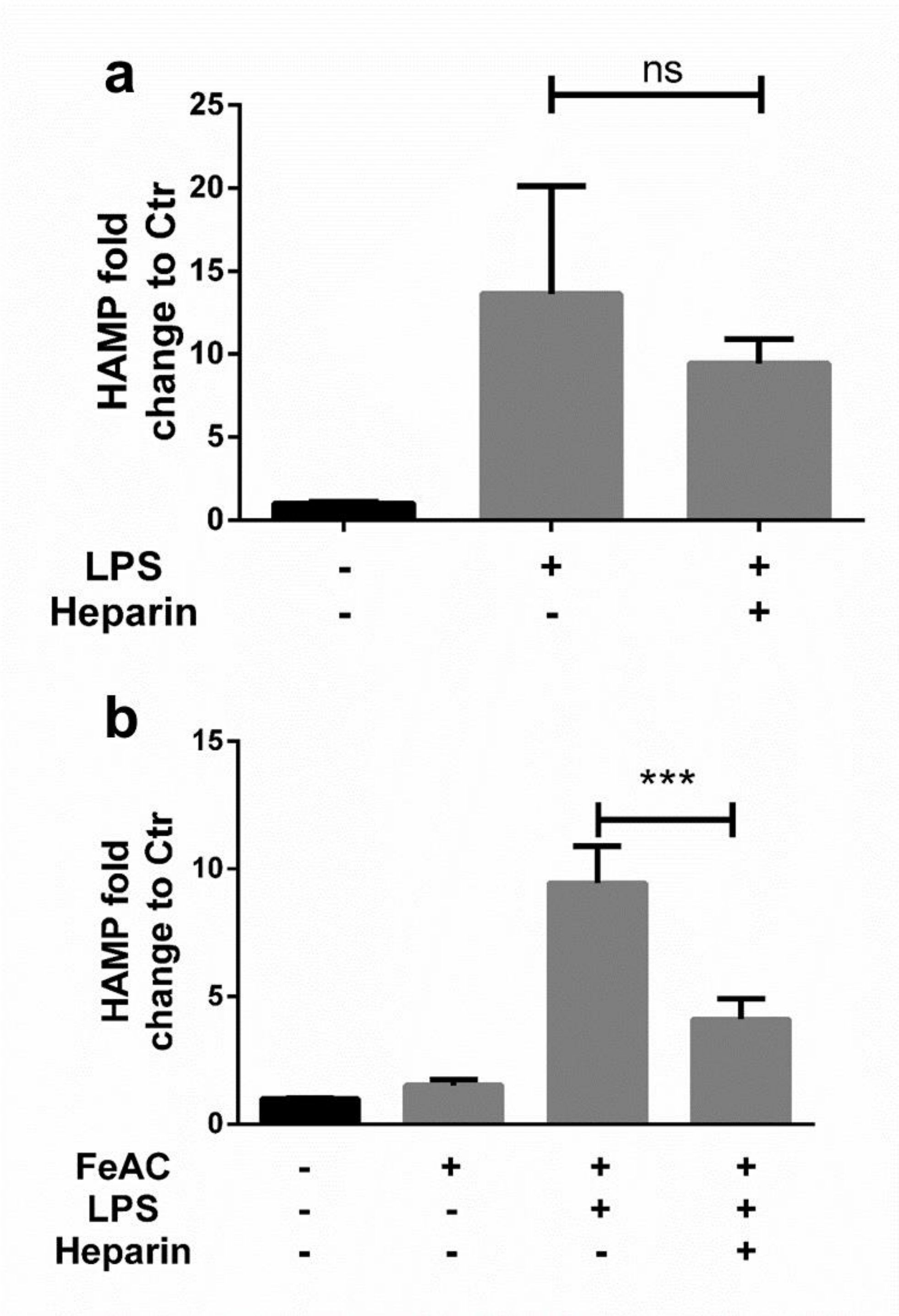
a
ns
HAMP fold change to Ctr
25
20
15
10
5
0
LPS - + +
Heparin - - +
b
15
***
HAMP fold change to Ctr
10
5
0
FeAC - + + +
LPS - - + +
Heparin - - - +

Fig S4

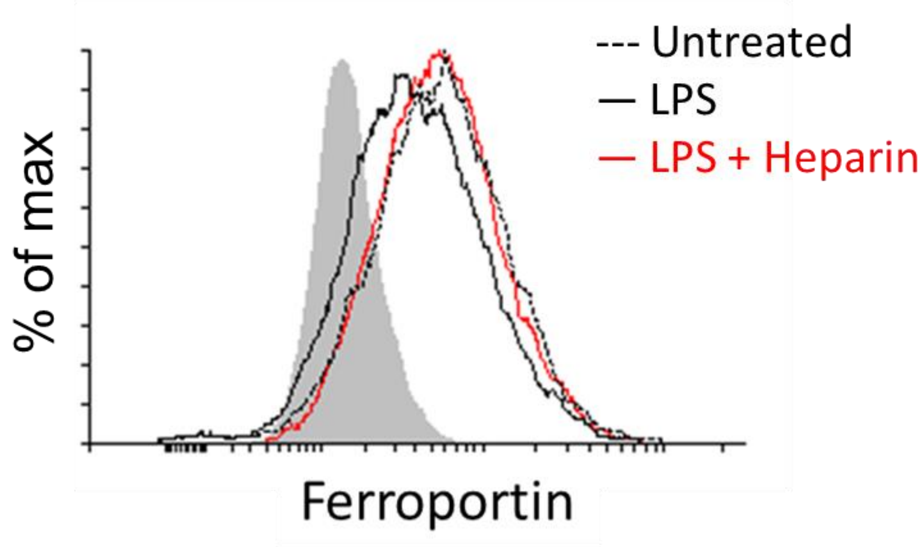

Fig S5

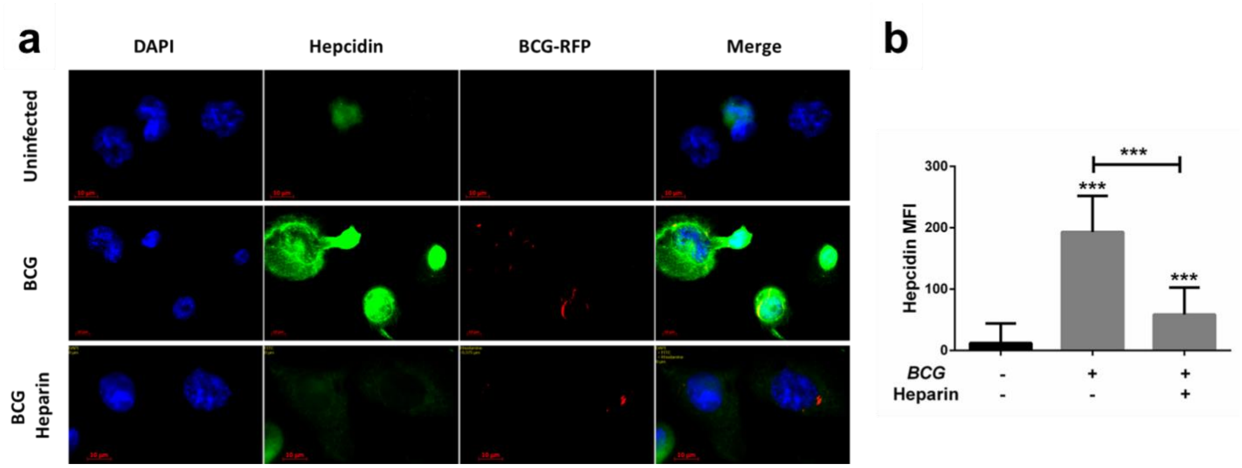
a
DAPI
Hepcidin
BCG-RFP
Merge
Uninfected
BCG
BCG Heparin
b
Hepcidin MFI
300
200
100
0
***
***
***
BCG
Heparin
-
-
+
-
+
+

Fig S6

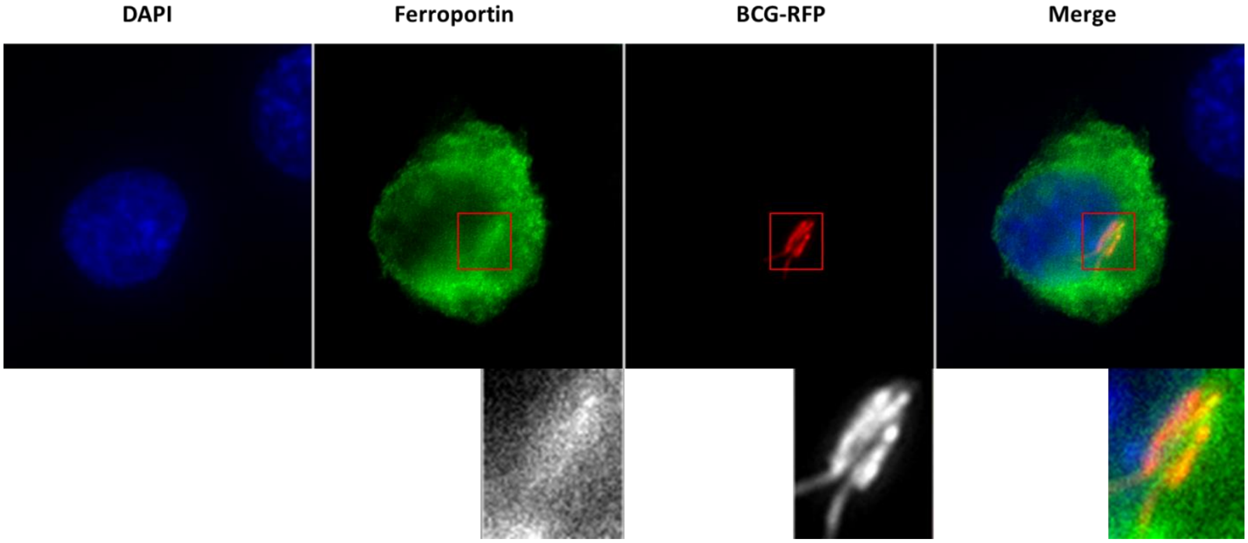

Fig S7

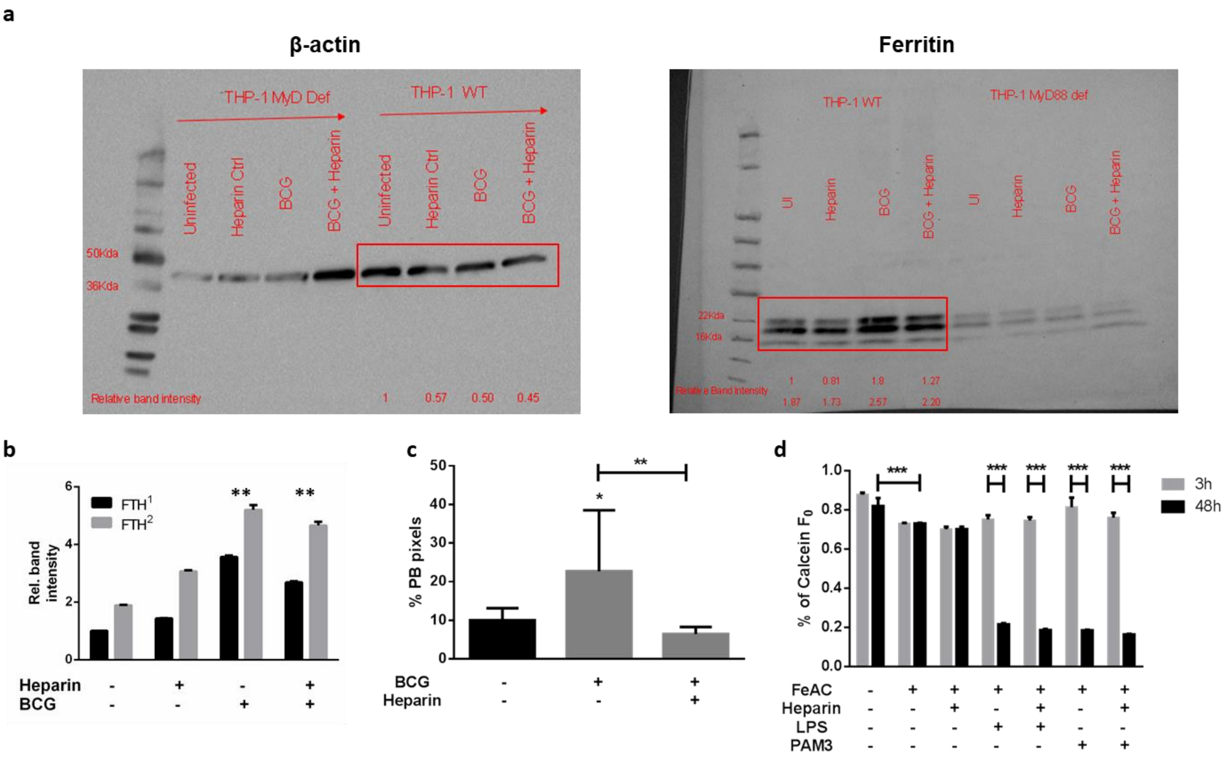
a
β-actin
THP-1 MyD Def
THP-1 WT
Uninfected
Heparin Ctrl
BCG
BCG + Heparin
50Kda
36Kda
Relative band intensity
1
0.57
0.50
0.45
Ferritin
THP-1 WT
THP-1 MyD88 def
UI
Heparin
BCG
BCG + Heparin
22Kda
16kda
Relative Band Intensity
1
0.81
1.8
1.27
1.87
1.73
2.57
2.20
b
Rel. band intensity
FTH1
FTH2
**
**
Heparin - + - +
BCG - - + +
c
% PB pixels
*
**
BCG - + +
Heparin - - +
d
% of Calcein F0
***
3h
48h
FeAC - + + + + + +
Heparin - - + - + - +
LPS - - - + + - -
PAM3 - - - - - + +
